# The efficacy and safety of traditional Chinese medicine's tonifying-kidney, strengthening-spleen, and invigorating-blood circulation (Bushen-Jianpi-Huoxue) principle for type 2 diabetes mellitus with osteoporosis: A protocol for systematic review and meta-analysis

**DOI:** 10.1097/MD.0000000000025197

**Published:** 2021-03-26

**Authors:** Yu Zhao, Jingjing Qiu, Tongying Chen, Shihao Wang, Shuhua Liu, Hongxing Huang, Lei Wan

**Affiliations:** a The Third Clinical Medical College; b Clinical Medical College of Acupuncture, Moxibustion and Rehabilitation, Guangzhou University of Chinese Medicine; c The Third Affiliated Hospital of Guangzhou University of Chinese Medicine, Guangzhou, PR China.

**Keywords:** Bushen-Jianpi-Huoxue principle, meta-analysis, protocol, systematic review, traditional Chinese medicine, type 2 diabetes with osteoporosis

## Abstract

**Background:**

Type 2 diabetes mellitus (T2D) and osteoporosis (OP) often coexist, and both are systemic metabolic diseases and seriously increase the risk of fragility fracture. However, there is no specific Western medicine for the treatment of T2D with OP (T2DOP). As reported in clinical and experimental studies, traditional Chinese medicine (TCM) based on principle of tonifying-kidney, strengthening-spleen, and invigorating blood circulation (Bushen-Jianpi-Huoxue) (BSJPHX) has significant efficacy against T2DOP. This protocol will be designed for a systematic review and meta-analysis to assess the efficacy and safety of TCM BSJPHX principle in the treatment of T2DOP.

**Methods:**

All relevant randomized controlled trials (RCTs) related to TCM therapies conducted in BSJPHX principle for T2DOP will be searched in the 8 electronic databases: PubMed, Cochrane Library, Wed of Science, EMBASE, Chinese National Knowledge Infrastructure Database (CNKI), Chinese Biomedical Literatures Database (CBM), Chinese Scientific Journal Database (VIP), Wanfang Database, from inception to October 2020. The main outcomes will contain: fasting blood glucose (FPG), 2 hours postprandial blood glucose (2hPG), glycosylated hemoglobin (HbA1c), serum calcium, bone mineral density (BMD), TCM syndrome integral, and the additional outcomes will consist of visual analog scale (VAS), and adverse events. Two reviewers will independently carry out literature search, data selection and synthesis, and literature quality assessment. In case of any dispute, it will be settled by group discussion. Assessment of risk of bias, reporting bias, and data synthesis would be performed with Review Manager software (Rev-Man 5.3).

**Result:**

This study will collate and summarize the various current evidences of TCM BSJPHX principle for T2DOP.

**Conclusion:**

This study will offer convincing evidence for judging the efficacy and safety of TCM BSJPHX principle for T2DOP.

**PROSPERO registration number:**

CRD42020218877.

## 1 Introduction

Diabetes mellitus (DM) is a chronic disease associated with severe complications.^[1]^ Type 2 diabetes mellitus (T2D) accounts for 90% of all DM.^[2]^ T2D is often accompanied by multiple complications, such as nephropathy, cardiovascular disease, neuropathy, retinopathy.^[3]^ Substantial evidence suggested that bone fragility should also be considered as one of the complications associated with T2D.^[1]^ In a systematic review that included 836,941 participants and 139,531 fracture cases, the risk of hip fracture was two- to three-fold higher in people with T2D than in people without diabetes, regardless of males or females.^[4]^ Clinical and epidemiological evidence indicate that there was an association between T2D and osteoporosis (OP).^[5]^ T2D and OP often coexist, and both are influenced by aging.^[6]^ A study claimed that 20% to 60% of patients with T2D developed OP.^[7]^ The diagnostic criteria for OP were based on the presence of low bone mineral density (BMD) and/or fragility fractures.^[8]^ OP is a systemic bone disease characterized by decreased bone mass and damage to bone microarchitecture, leading to increased bone fragility and susceptibility to fracture.^[8]^ According to the study statistics, 30% of women and 20% of men older than 50 years experience an osteoporotic fracture at some point in their lifetime.^[9]^ With more than 9 million osteoporotic fractures occurring annually worldwide, OP is an important cause of morbidity and life loss years globally, accounting for approximately 0.83% of the global burden of noncommunicable diseases (NCDs) on the basis of disability adjusted life years (DALY).^[10]^ Fragility fractures prevention is the main treatment goal of OPs.^[11]^

It is well known that lifestyle interventions are the basis of treatment for patients with T2D, but most will ultimately require pharmacological treatment. There are many drugs available for the treatment of T2D, but they have different effects on bone metabolism and the risk of fragility fractures.^[6]^ The treatment of Western medicine for T2D with OP (T2DOP) is generally based on the control of T2D and the addition of drugs to relieve OP, but it is difficult to achieve satisfactory curative effect. In addition, only denosumab, bisphosphonates, teriparatide, strontium raninate, and selective estrogen receptor modulators (raloxifene and bazedoxifene) are currently approved drugs for the treatment of OP. However, data on their effects on glycemia in patients with T2D are scarce, as are data on their effects on glucose metabolism in patients without T2D.^[6]^

In traditional Chinese medicine (TCM), T2D is roughly equivalent to “thirst wasting (Xiaoke)” and OP is similar to “bone impotence (Guwei).”^[12]^ The TCM mechanism of T2DOP is based on the deficiency of spleen and kidney as the root cause, and stasis blood blocking collaterals as the surface cause.^[13]^ TCM has the characteristics of good overall effect, relatively low price and few adverse reactions for the prevention and treatment of T2DOP.^[14]^ A review study found that some single herbs such as longspur epimedium and prepared rehmannia glutinosa had the efficacy to tonify the kidney and represented the potential to cure T2DOP. Compared with Western medicines, these medicines have fewer side effects, lower prices, and relatively stable and long-lasting efficacy. And TCM believes that “nonunion will not pain,” if the herbs of invigorating blood circulation and eliminating stasis are added at the same time, it shows specific advantages for alleviating the pain triggered by OP.^[15]^ Therefore, for the treatment of T2DOP, the TCM strategy should be based on tonifying-kidney, strengthening-spleen, and invigorating blood circulation (Bushen-Jianpi-Huoxue) (BSJPHX) principle.

## 2 Methods

### 2.1 Registration

The protocol has been registered at PROSPERO (registration No. CRD42020218877). This study would be conducted in compliance with the preferred reporting items for systematic reviews and meta-analyses (PRISMA) statement guidelines^[16]^ and in conformity to the Cochrane Handbook for Systematic Reviews of Interventions.^[17]^

### 2.2 Type of studies

To evaluate the efficacy and safety of traditional Chinese medicine BSJPHX principle for T2DOP, all randomized controlled trials (RCTs) regardless of the blind method and language which explored the specific effect of TCM therapies based on BSJPHX principle in the treatment of T2DOP will be included. Animal experiments, conference papers, reviews, case reports, and duplicate publications or literatures lacking data will be excluded.

### 2.3 Types of participants

Participants in those included RCTs must be diagnosed with T2D and OP (reference to any authoritative and recognized diagnostic criteria). There is no restriction on gender, race, or nation. Patients accompanied by other metabolic diseases will be excluded.

### 2.4 Types of interventions

A kind of TCM treatment methods as the individual intervention or a core part of combination therapy with conventional Western medicines. Specific TCM treatment methods consisting of traditional Chinese herbal medicine, acupuncture, moxibustion, Chinese massage, acupoint injection, or acupoint catgut embedding must be based on BSJPHX principle.

### 2.5 Types of comparisons

The control group will include blank control or placebo control. If TCM therapies are combined with Western medicine in the experimental group, the control group must use the same medicine in the same way.

### 2.6 Types of outcome measures

1. The primary outcomes will include 2 parts:
  a. Outcome indicators of Western medicine: fasting blood glucose (FPG), 2 h postprandial blood glucose (2hPG), glycosylated hemoglobin (HbA1c), serum calcium, BMD.
  b. Outcome indicators of TCM: TCM syndrome integral before and after treatment based on *Guiding principles for clinical research of new drugs of TCM.*^[18]^
2. The secondary outcomes will include visual analog scale (VAS), and adverse events (fever, diarrhea, infection, bruising, numbness, local pain, and so on).

### 2.7 Literature search strategy

We will comprehensively search 4 international electronic databases containing PubMed, EMBASE, Cochrane Library and Web of Science and 4 electronic Chinese databases containing Chinese National Knowledge Infrastructure (CNKI), Chinese Biomedical and Medical Database (CBM), Wan Fang Database and Chinese Scientific Journal Database (VIP) with a time span from their initiation to October 2020. The search strategy was constructed around search terms for BSJPHX principle, T2DOP, and RCTs and applied for all databases. The search strategy in PubMed will be as follows: ((Bushen-Jianpi-Huoxue) OR (tonifying-kidney, strengthening-spleen and invigorating blood circulation)) AND ((Type 2 diabetes) AND (osteoporosis)) AND (randomized controlled trial). Chinese translation of key words will be used in the search strategy of Chinese databases: ((bu shen jian pi huo xue)) AND (((2 xing tang niao bing) OR (xiao ke)) AND ((gu zhi shu song zheng) OR (gu wei) OR (gu ku) OR (gu bi)) AND ((sui ji dui zhao shi yan) OR (sui ji) OR (dui zhao) OR (lin chuang) OR (liao xiao)). The included RCTs will be screened for further materials.

### 2.8 Data selection

**Figure 1:**
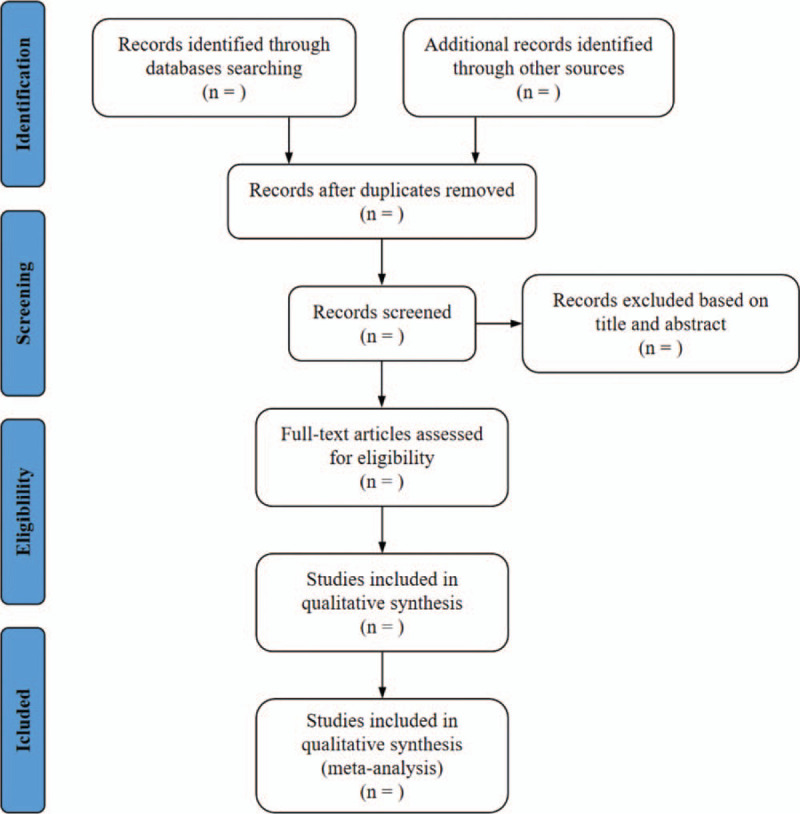
Flow diagram of study selection process.

In the data selection, 2 reviewers (ZY and QJJ) will independently carry out the literature search strategy. After literature search, the 2 reviewers will screen the retrieved articles based on reading the titles and the abstracts and reviewed full text after preliminary screening to remove and reserve studies in accordance with the inclusion criteria and exclusion criteria. If disagreements arose, the third researcher (CTY) would join discussions to ensure consistency of all included studies. The study selection process will be displayed through the PRISMA flow chart (Fig. 1).

The list of extracted data will include the first author, year of publication, diagnostic criteria, sample size, duration of T2D/OP, intervention methods, details of interventions, characteristics of patients in each group, outcomes, *P* value, adverse events, withdrawal and follow up, among other factors.

### 2.9 Assessment of risk of bias

Two reviewers (ZY and QJJ) will evaluate the quality of each included RCTs in strict accordance with the Cochrane handbook-bias risk assessment tool.^[17]^ Additionally, disagreements will be solved through discussion among all authors. The risk of bias will be assessed from the 7 items: selection bias (random sequence generation), selection bias (allocation concealment), performance bias (binding of participants and personnel), detection bias (binding of outcome assessment), attrition bias (incomplete outcome data), reporting bias (selective reporting), and potential bias (other bias). Each item will be classified as follows: L (low risk), H (high risk), and U (unclear risk). For the evaluation of the overall quality, an RCT will be classified as “L” if more than 3 of its items are judged as “L.” And it will be classified as “H” if more than 3 of its items are evaluated as “H” or “U.” In other cases, it will be classified as “U.”

### 2.10 Data synthesis

#### 2.10.1 Statistical analysis and data synthesis

Cochrane Collaboration Review Manager software (Rev-Man 5.3) will be used to perform the meta statistical analysis. With 95% confidence intervals (CIs), mean difference (MD) or standardized mean difference (SMD) will be used for continuous outcomes, and risk ratio (RR) will be applied to dichotomous outcomes. In the aspect of data synthesis, *I*^2^ test statistic will be used to evaluate the heterogeneity of the included RCTs. If *I*^2^ < 50% and *P* > .1, the outcome will indicate no homogeneity in the studies and the fixed effect (FE) model will be adopted for analysis. Otherwise, the outcome will be of heterogeneity and the source of heterogeneity will require further analysis. The random effect (RE) model will be adopted to the meta-analysis after getting rid of the influence of clinical heterogeneity.

#### 2.10.2 Subgroup analysis

We will perform some planned subgroup analysis: different treatment methods of TCM (traditional Chinese herbal medicine, acupuncture, moxibustion, Chinese massage, acupoint injection, and acupoint catgut embedding), and different treatment periods of the BSJPHX principle and different follow-up periods which will be set according to the included literatures.

#### 2.10.3 Sensitivity analysis and reporting bias analysis

By excluding low-quality studies, sensitivity analysis will be conducted to determine the robustness and stability of the combined results. The main result (the efficiency of different TCM methods under the guidance of BSJPHX principle) in included RCTs will be made into a funnel chart to evaluate the bias of the report.

#### 2.10.4 Quality of evidence

Under the guidance of Grading of Recommendations Assessment, Development, and Evaluation (GRADE) system, the quality of evidence for the outcomes will be assessed. The 5 evaluation items include: limitations, inconsistency, indirectness, imprecision, and publication bias. Quality of evidence will be rated in 4 levels: high, moderate, low, and very low.

### 2.11 Ethics and dissemination

This study will not require ethical approval because the data extracted from the included studies will not involve personal information of patients and will not lead to violations of participants’ privacy. Our goal is to publish this systematic review in a peer-reviewed journal.

## 3 Discussion

Both T2D and OP are affected by aging and lifestyle changes. These 2 diseases often coexist, but the Western medications used in each disease affect the progress of the other.^[6]^ TCM believes that deficiency of kidney and spleen, vein stasis, and bone marrow dystrophy are the basic pathogenesis of T2D and OP. Therefore, many researchers have done a lot of experimental or clinical studies on TCM BSJPHX principle for T2DOP. A study^[19]^ proved through experiments that BSJPHX decoction can decrease fasting blood glucose, insulin resistance and increase BMD in T2DOP rats. And it attenuates T2DOP by activating Wnt signaling pathway and inhibiting NF-κB signaling pathway. Wu^[20]^ found that BSJPHX principle could significantly improve the clinical symptoms of TCM in patients with T2DOP, effectively reduce blood glucose and glycosylated hemoglobin, fasting insulin level and insulin resistance index, and improve BMD and serum IGF-1, IRS-1, IRS-2 levels.

The combination of all the methods (tonifying kidney to fill essence and marrow, invigorating spleen to nourish Qi and blood, activating blood to dredge meridians) and the consideration of the specimen can not only enhance the function of viscera and the metabolism of water and grain essence (Xiaoke), but also improve the nutrition of muscles and bones and alleviate the state of osteoporosis (Guwei).

To our knowledge, the review will be the first systematic review and meta-analysis of TCM BSJPHX principle for T2DOP. It will summarize the data to prove the effectiveness and safety of BSJPHX principle for T2DOP, provide reliable guidance for clinical use of BSJPHX principle for T2DOP, and may provide some alternative and complementary therapies based on the principle for policy makers to reduce the burden of public health.

## Author contributions

**Conceptualization:** Yu Zhao, Jingjing Qiu, Tongying Chen, Shihao Wang, Shuhua Liu, Hongxing Huang, Lei Wan.

**Data curation:** Yu Zhao, Jingjing Qiu, Tongying Chen.

**Formal analysis:** Yu Zhao, Jingjing Qiu, Tongying Chen.

**Methodology:** Yu Zhao, Jingjing Qiu, Tongying Chen.

**Project administration:** Hongxing Huang, Lei Wan.

**Resources:** Yu Zhao, Jingjing Qiu, Tongying Chen.

**Software:** Yu Zhao, Jingjing Qiu, Shihao Wang.

**Visualization:** Yu Zhao, Jingjing Qiu, Shuhua Liu.

**Writing – original draft:** Yu Zhao, Jingjing Qiu, Tongying Chen.

**Writing – review & editing:** Yu Zhao, Jingjing Qiu, Lei Wan.
